# Distribution pattern of left ventricular myocardial strain by feature-tracking CMR in Chinese normal subjects

**DOI:** 10.1186/1532-429X-18-S1-P34

**Published:** 2016-01-27

**Authors:** Hong Liu, Dan Yang, Yong Luo, Ke Wan, Tianjing Zhang, Yucheng Chen

**Affiliations:** 1 grid.412901.f0000000417701022Cardiology Department, West China Hospital Sichuan University, Chengdu, China; 2 Siemens Healthcare, Beijing, China

## Background

Myocardial strain was regarded as a sensitive parameter to quantify left ventricular function. Feature tracking CMR is emerging as a new simple and reliable technique to evaluate myocardial strain. In the present study, we were sought to investigate the distribution characteristics of left myocardial strain on the transmural direction or longitudinal direction in healthy Chinese subjects.

## Methods

A total of 130 healthy Chinese subjects were studied at 3T MRI scanner (Tim Trio, Siemens, Germany). A stack of short axis slices covering the whole left ventricle and standard 2/3/4 chamber cines were acquired by SSFP sequence. 3 dimensional strains (radial, circumferential and longitudinal) of left ventricle were analyzed by using feature-tracking CMR technique (Trufisp Strain software, Siemens, Germany). Transmural distribution pattern of laminar radial and circumferential strain (endo/mid/epicardial) was analyzed, and longitudinal distribution pattern (from basal to apex) of strains was also analyzed.

## Results

A transmural gradient existed in circumferential strain, which showed an universal increment trend from endocardial wall to epicardial myocardial wall in basal, mid and distal short axis slices (basal [endo: -21.44 ± 2.16%; mid: -18.06 ± 1.9%; epi: -14.59 ± 1.81%; P < 0.001 ]; mid [endo: -20.35 ± 2.42%; mid: -17.05 ± 1.99%; epi: -13.76 ± 1.71%; P < 0.001]; apical [endo: -25.02 ± 3.76%; mid: -21.42 ± 3.66%; epi: -17.81 ± 3.62%; P < 0.001]). However, the transmural distribution of radial strain was heterogeneous. In mid slices, midcardial is higher in comparison with endocardial and epicardial (endo: 40.65 ± 9.18%; mid: 46.01 ± 9.59%; epi: 44 ± 8.67%; P < 0.001), but the same trend of circumferential strain can be seen in basal (endo: 31.33 ± 8.41%; mid: 41.9 ± 9.47%; epi: 46.29 ± 9.72%; P < 0.001) and distal (endo: 30.76 ± 11.39%; mid: 37.17 ± 11.7%; epi: 38.9 ± 10.94%; P < 0.001) slices. In longitudinal direction, peak systolic radial strain was higher in mid LV slice than in basal or distal slice (basal: 38.81 ± 8.52%; mid: 42.45 ± 8.58%; apical: 35.18 ± 10.63%; P < 0.001). Peak circumferential strain of mid-LV is the lowest in three LV slices (basal: -17.59 ± 1.81%; mid: -16.84 ± 1.87%; apical: -21.03 ± 3.61%; P < 0.001). As for longitudinal strain, there was decreasing trend from base to apex, either in septal (basal: -23.3 ± 4.65%; mid: -13.66 ± 7.33%; apical: -13.24 ± 5.51%; P < 0.001) or lateral myocardial wall (basal: -24.67 ± 5.62%; mid: -15.43 ± 5.85%; apical: -12.96 ± 4.26%; P < 0.001).

## Conclusions

A transmural gradient of circumferential strain within LV myocardial wall existed in Chinese normal subjects. Longitudinal strain also showed a decreasing trend from basal to apex of LV. These distribution pattern of strains in healthy subjects may provide unique profiles for further study in different myocardial disease conditions.

